# Molecular signaling underlying bulleyaconitine A (BAA)-induced microglial expression of prodynorphin

**DOI:** 10.1038/srep45056

**Published:** 2017-03-22

**Authors:** Teng-Fei Li, Hai-Yun Wu, Yi-Rui Wang, Xin-Yan Li, Yong-Xiang Wang

**Affiliations:** 1 King’s Lab, Shanghai Jiao Tong University School of Pharmacy, 800 Dongchuan Road, Shanghai 200240, China

## Abstract

Bulleyaconitine (BAA) has been shown to possess antinociceptive activities by stimulation of dynorphin A release from spinal microglia. This study investigated its underlying signal transduction mechanisms. The data showed that (1) BAA treatment induced phosphorylation of CREB (rather than NF-κB) and prodynorphin expression in cultured primary microglia, and antiallodynia in neuropathy, which were totally inhibited by the CREB inhibitor KG-501; (2) BAA upregulated phosphorylation of p38 (but not ERK or JNK), and the p38 inhibitor SB203580 (but not ERK or JNK inhibitor) and p38β gene silencer siRNA/p38β (but not siRNA/p38α) completely blocked BAA-induced p38 phosphorylation and/or prodynorphin expression, and antiallodynia; (3) BAA stimulated cAMP production and PKA phosphorylation, and the adenylate cyclase inhibitor DDA and PKA inhibitor H-89 entirely antagonized BAA-induced prodynorphin expression and antiallodynia; (4) The Gs-protein inhibitor NF449 completely inhibited BAA-increased cAMP level, prodynorphin expression and antiallodynia, whereas the antagonists of noradrenergic, corticotrophin-releasing factor, A1 adenosine, formyl peptide, D1/D2 dopamine, and glucagon like-peptide-1 receptors failed to block BAA-induced antiallodynia. The data indicate that BAA-induced microglial expression of prodynorphin is mediated by activation of the cAMP-PKA-p38β-CREB signaling pathway, suggesting that its possible target is a Gs-protein-coupled receptor – “aconitine receptor”, although the chemical identity is not illustrated.

The antinociceptive effects of the *Aconitum* extracts have been demonstrated in a variety of experimental pain studies, such as the tail-pressure, paw-pressure, tail-flick, and hot-plate tests, acetic acid writhing, repeated cold stress pain, adjuvant-induced arthritic pain, streptozotocin-induced diabetes pain, and peripheral nerve injury-induced neuropathic pain^1^,^2^,^3^,^4^,^5^. It is evident that the antinociceptive effects of *Aconiti* were mediated by the pharmacological action of diterpenoid alkaloid and to date, there are approximately 170 diterpenoid alkaloids identified, including aconitine, bulleyaconitine A, mesaconitine, hypaconitine, bullatine A, and lappaconitine, which could be mainly classified into three skeletal categories, i.e., C_18_-, C_19_-, and C_20_-diterpenoid alkaloids^6^,^7^. Bulleyaconitine A (BAA), a C19-diterpenoid alkaloid isolated from *Aconitum bulleyanum*, is marketed in China to treat chronic pain and rheumatoid arthritis^8^. Earlier studies demonstrated that systemic BAA administration blocked acetic acid-induced writhing response and formalin-induced tonic hyperalgesia^8^. A recent study further demonstrated that subcutaneous and intrathecal administration of BAA and its analog aconitine markedly attenuated peripheral spinal nerve ligation- and paclitaxel-induce neuropathic pain and cancer cell inoculation-induced bone pain, in addition to its inhibition of formalin-induced tonic pain, although they did not reduce formalin-induce acute flinching response^9^,^10^. Moreover, bullatine A, a C_20_-diterpenoid alkaloid, significantly attenuated pain hypersensitivity, including mechanical allodynia and thermal hyperalgesia in a variety of rodent models of pain, such as peripheral neuropathy, bone cancer pain, streptozocin diabetes-induced pain, formalin-induced tonic pain, and complete Freud’s adjuvant-induced rheumatic inflammatory pain^11^. The antinociceptive activity was also demonstrated in lappaconitine, a C18-diterpenoid alkaloid, in neuropathy and bone cancer pain^12^.

It has been generally accepted that interaction of BAA and their analogs with neuronal voltage-gated sodium channels promoted aconitines antinociception and mediated their toxicity^7^,^13^,^14^, but a novel analgesic mechanism involving release of spinal microglial dynorphin A and subsequent activation of neuronal κ-opioid receptors have been also recently proposed^9^,^10^,^11^. It was initially discovered by a Japanese group showing that the processed Aconiti tuber antinociception in a mouse nociceptive model of repeated cold stress was eliminated by intrathecal injection of the dynorphin A antibody and the κ-opioid receptor antagonist NBI^3^,^4^,^5^. The King’s Lab in China further confirmed that (i) the dynorphin A antibody and κ-opioid receptor antagonism (NBI and GNTI) in neuropathy eliminated antinociceptive effects of the purified active ingredients and their metabolites of *Aconiti*, including BAA^9^, bullatine A^11^, lappaconitine^12^, aconitine and benzoylaconine^10^; (ii) the active ingredients and their metabolites of *Aconiti* could stimulate dynorphin A expression and secretion from the spinal cords of neuropathic rats^9^,^10^,^11^; (iii) The stimulated dynorphin A expression was specifically derived from spinal microglia but not neurons or astrocytes, identified by the cellular chemistry assay, double immunofluorescence staining technique, and application of the microglia inhibitor minocycline. However, activation of spinal microglia was not required for aconitines to express dynorphin A, as aconitines, for similar degrees, stimulated dynorphin A expression in bath contralateral/sham and ipsilateral spinal dorsal horn, and cultured primary microglia both in the presence and absence of lipopolysaccharides treatment^9^,^10^,^11^,^12^; (iv) Aconitines antinociception was separated from neurotoxicity, demonstrated by the inability of the sodium channel blockers lidocaine and ropivacaine on dynorphin A expression and BAA-stimulated dynorphin A expression, and inability of minocycline on BAA-induced acute neurotoxicity^9^. Taken together, all these data suggest that aconitines, including BAA, produced antinociception in pain hypersensitivity states specifically through dynorphin A expression and secretion from spinal microglia, but not through their counteractions with neuronal voltage-gated sodium channels. It is known that dynorphin A is an endogenous κ-opioid receptor agonist, distributes throughout the central nervous system, localized and secreted in neurons, astrocytes, and microglia^9^,^15^,^16^, and serves multiple regulatory functions, such as analgesia, psychomimesis, dysphoria, diuresis, and antipruritic effects^17^, in the central nervous system.

G-protein-coupled receptors (GPCRs) constitute the largest family of cell surface proteins for cell signal transduction. G-proteins are heterotrimeric signaling molecules composed of α, β and γ subunits, and are divided into four major classes, i.e., Gs, Gi, Gq and G_12_, on the basis of amino acid similarities of their α-subunits^18^. Although such a classification is rather arbitrary, there is a general mechanism among the members of the subfamily. Stimulation of the Gs subfamily activates adenylyl cyclase and stimulates accumulation of a diffusible second messenger cyclic AMP (cAMP) and activation of cAMP-activated protein kinase A (PKA)^19^. The activated form of PKA in turn induces phosphorylation of a large variety of the downstream target proteins, including transcription factors like the cAMP response element-binding protein (CREB) and NF-κB, to ultimately regulate numerous cell events^20^. Serine 133 is a well-characterized CREB phosphorylation site and can be phosphorylated by PKA and the cAMP-PKA/CREB signaling can be initiated as a classical pathway^21^. An alternative pathway of the mitogen-activated protein kinase (MAPK)/CREB is also later proposed after the observation that CREB phosphorylation can also be induced by p38 MAPK on the same serine 133 site^22^. It is known that CREB is able to induce the transcription of the dynorphin A precursor prodynorphin gene in neurons^22^,^23^. Therefore, this study aimed to systemically investigate signal transduction pathways involving CREB activation for aconitines represented by BAA to upregulate prodynorphin expression in microglia.

## Results

### CREB activation mediates BAA-induced prodynorphin expression in primary microglia

**Figure 1:**
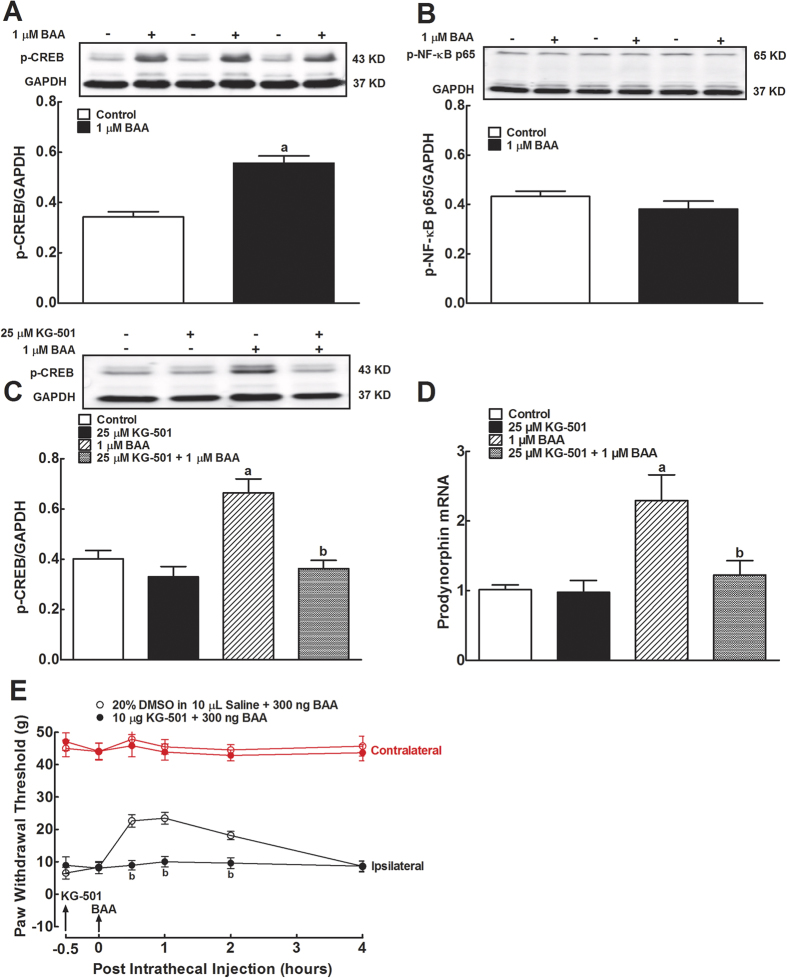
Effects of bulleyaconitine A (BAA) on phosphorylation of CREB (**A**) and NF-κB (**B**), and blockade effects of the specific CREB activation inhibitor KG-501 on BAA-induced CREB phosphorylation (**C**) and prodynorphin expression (**D**) in primary cultures of microglia, and spinal mechanical antiallodynia (**E**) in neuropathic rats. Primary microglial cells were collected from the spinal cord of 1-day-old neonatal rats. For the CREB and NF-κB phosphorylation, 1 μM BAA was added into primary microglia culture for 1 hour and the levels of phosphorylated CREB and NF-κB p65 were determined using Western blot. The representative blots are shown in the upper panels of each panel. The dynorphin A precursor prodynorphin mRNA expression was measured using qRT-PCR and normalized to *GAPDH* mRNA. For mechanical antiallodynia, neuropathic rats were induced by tight ligation of L5/L6 spinal nerves and mechanical thresholds were measured in both contralateral and ipsilateral paws. Data are expressed as means ± SEM (n = 3 in each treatment with two independent repeats in cultured primary microglial cells, and n = 6 in each group of neuropathic rats). ^a,b^*P* < 0.05 using the unpaired and two-tailed Student’s *t*-test or two-way repeated measures ANOVA followed by the post-hoc student-Newman-Keuls test, compared with the control and BAA groups, respectively.

We first assessed the stimulatory effects of BAA on phosphorylation of the transcription factors CREB and NF-κB in primary cultures of microglia. Incubation of 1 μM of BAA for 1 hour induced a robust increase in CREB phosphorylation by 62%, measured by Western blot, compared to the control group (*P* < 0.05 using unpaired and two-tailed Student’s *t*-test; Fig. 1A). In contrast, the same BAA concentration did not significantly affect NF-κB p65 phosphorylation (Fig. 1B).

Pretreatment with the specific CREB activation inhibitor KG-501 (25 μM)^24^ for 30 minutes did not significantly affect the basal CREB phosphorylation level, whereas BAA treatment (1 μM) induced CREB phosphorylation by 66%, which was completely inhibited by 30 minute-KG-501 pretreatment (*P* < 0.05 by two-way ANOVA followed by the post-hoc Student-Newman-Keuls test; Fig. 1C).

Since BAA-increased dynorphin A level measured by the enzyme-linked immunosorbent assay was in parallel with the expression of the cellular prodynorphin gene encoding dynorphin A in spinal microglia from neonatal and adult rats^9^, prodynorphin expression was only measured in this study. Indeed, BAA incubation (1 μM) of cultured primary microglial cells for 2 hour significantly increased expression of prodynorphin mRNA by 2.3 fold, measured by qRT-PCR. The stimulatory effect of BAA was completely inhibited by KG-501 (25 μM) pretreatment, although it did not significantly alter the basal prodynorphin expression (*P* < 0.05 using two-way ANOVA followed by the post-hoc Student-Newman-Keuls test; Fig. 1D).

Furthermore, KG-501 was also given to neuropathic rats induced by tight ligation of spinal nerves. Intrathecal injection of normal saline (10 μL) or KG-501 (10 μg)^25^ did not significantly affect baseline paw withdrawal thresholds in response to mechanical stimulation in both contralateral and ipsilateral paws, whereas intrathecal BAA (300 ng) markedly and time-dependently increased mechanical thresholds in the ipsilateral paws (but not in the contralateral paws) of saline-pretreated rats. BAA mechanical antiallodynia was totally suppressed by 30 minute-pretreatment of KG-501 (*P* < 0.05 using two-way ANOVA followed by a post-hoc Student-Newman-Keuls test; Fig. 1E).

### p38 activation mediates BAA-induced prodynorphin expression in primary microglia

**Figure 2:**
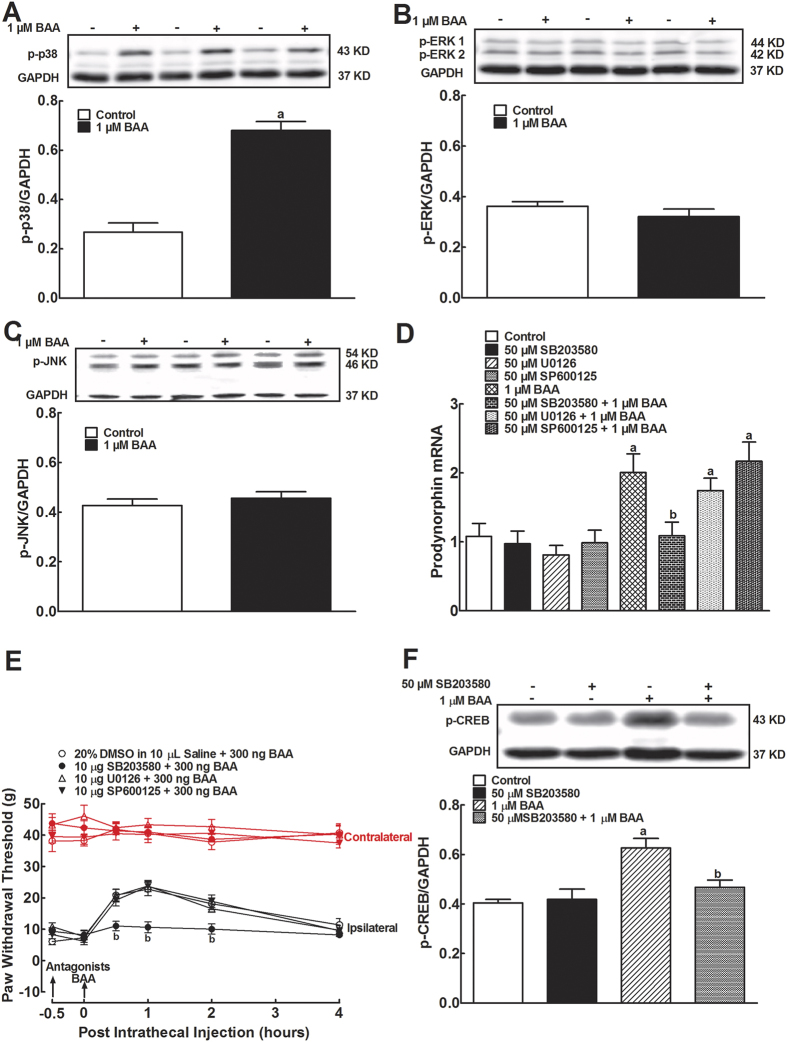
Effects of bulleyaconitine A (BAA) on levels of phosphorylated p38 (**A**), ERK (**B**) and JNK MAPK (**C**), and blockade effects of the specific inhibitors of p38, ERK and JNK on BAA-induced expression of prodynorphin mRNA level. (**D**) in primary cultures of microglia, and spinal mechanical antiallodynia in neuropathic rats (**E**). Primary microglial cells were collected from the spinal cord of 1-day-old neonatal rats. For phosphorylation, 1 μM BAA was added into microglia culture for 1 hour and levels of phosphorylated p38, ERK, JNK and CREB were determined by using Western blot. The representative blots are shown in the upper panels of each panel. For gene expression, the MAPK inhibitors were incubated 30 minutes before BAA treatment and primary microglial cells were collected 2 hours later. Expression of the dynorphin A precursor prodynorphin gene was determined using qRT-PCR and normalized to *GAPDH* mRNA. For mechanical antiallodynia, neuropathic rats were induced by tight ligation of L5/L6 spinal nerves, and mechanical thresholds were measured in both contralateral and ipsilateral paws. (**F**) Blockade effect of the p38 inhibitor SB203580 on BAA-induced CREB phosphorylation in cultured primary microglia. Data are expressed as means ± SEM (n = 3 in each treatment with two independent repeats in cultured primary microglial cells, and n = 6 in each group of neuropathic rats). ^a,b^*P* < 0.05 using the unpaired and two-tailed Student’s *t*-test or two-way repeated measures ANOVA followed by the post-hoc student-Newman-Keuls test, compared with the control and BAA groups, respectively.

MAPKs are a family of signaling molecules to transduce extracellular stimuli into intracellular responses in a wide variety of circumstances. The MAPK family includes p38, ERK and JNK, and is activated through different upstream transduction chain reactions^26^. In order to assess whether BAA affected MAPK activation, we measured phosphorylation of p38, ERK and JNK using Western blot. Treatment with 1 μM BAA in cultured primary microglia for 1 hour significantly induced p38 phosphorylation by 2.5 fold (*P* < 0.05 using the unpaired and two-tailed Student’s *t*-test; Fig. 2A). However, the same concentration of BAA failed to activate ERK (Fig. 2B) or JNK (Fig. 2C).

To further determine whether p38 activation was causally associated with BAA-stimulated prodynorphin expression, the selective p38 inhibitor SB203580, ERK inhibitor U0126, and JNK inhibitor SP600125 were employed. SB203580 (50 μM)^27^, U0126 (50 μM)^28^,^29^ and SP600125 (50 μM)^30^ treatment did not significantly alter basal levels of prodynorphin expression, whereas 30 minutes SB203580 pretreatment completely blocked 1 μM BAA-increased prodynorphin expression (*P* < 0.05 using two-way ANOVA followed by a post-hoc Student-Newman-Keuls test). In contrast, neither SP600125 nor U0126 significantly suppressed BAA-increased prodynorphin expression (Fig. 2D).

In neuropathic rats, mechanical thresholds were measured in both contralateral and ipsilateral paws. Intrathecal injection of 10 μL of normal saline or 10 μg each of SB203580, U0126 and SP600125^31^,^32^,^33^ did not significantly affect baseline mechanical withdrawal response in either contralateral or ipsilateral paws. In saline-pretreated rats, intrathecal BAA (300 ng) in the ipsilateral paws produced a time-dependent mechanical antiallodynia, which was completely prevented by the 30 minutes SB203580 pretreatment (*P* < 0.05 using two-way ANOVA followed by a post-hoc Student-Newman-Keuls test), but not with U0126 and SP600125 (Fig. 2E).

We further assessed the p38-dependency of BAA-induced CREB phosphorylation. Treatment with SB203580 (50 μM) did not significantly affect the basal levels of CREB phosphorylation, but nearly completely inhibited BAA (1 μM)-upregulated CREB phosphorylation (*P* < 0.05 using two-way ANOVA followed by the post-hoc Student-Newman-Keuls test; Fig. 2F).

### p38β phosphorylation mediates BAA-induced prodynorphin expression in spinal microglia

**Figure 3:**
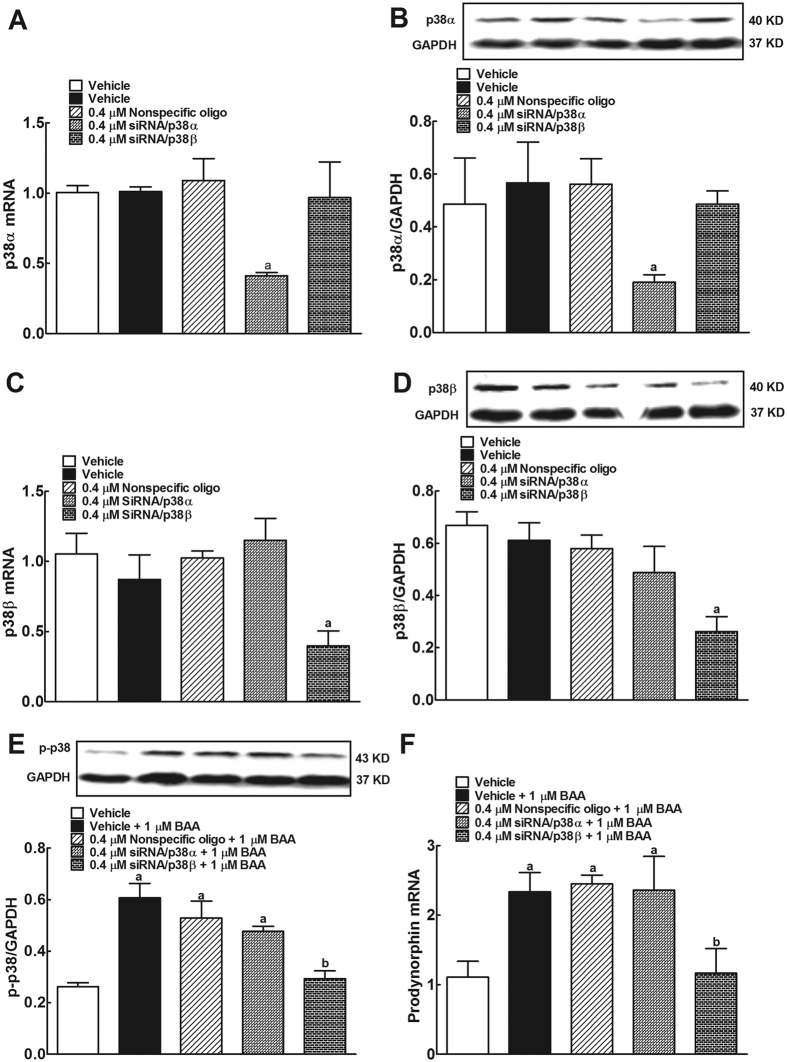
Differential effects of siRNA/p38α and siRNA/p38β on expression of p38α (**A**,**B**) and p38β mRNA and protein (**C**,**D**), bulleyaconitine A-induced p38 phosphorylation (**E**) and prodynorphin gene expression (**F**) in primary cultures of microglia. Primary microglial cells were collected from the spinal cord of 1-day-old neonatal rats. For the gene knockdown, siRNA/p38α and siRNA/p38β were transfected into primary microglia for 5 hours and microglia were cultured for additional 24 hours after refreshed the culture medium. Expressions of the mRNA of p38α, p38β and prodynorphin and protein of p38α and p38β referred to *gapdh* were determined using qRT-PCR and Western blot, respectively. For the phosphorylation assay, primary microglial cells were treated with 1 μM BAA for 1 hour and then subjected to Western blot analysis of phosphorylated p38 with an anti-phospho-p38 antibody. The representative blots are shown in the upper panels of each panel. Data are expressed as means ± SEM (n = 3 per group in each treatment with three independent repeats). ^a,b^*P* < 0.05 using one-way or two-way repeated measures ANOVA followed by the post-hoc student-Newman-Keuls test, compared with the nonspecific oligonucleotide control and BAA groups, respectively.

There are four isoforms of p38 MAPK, i.e., α, β, γ, and δ, among which α and β are the major isoforms in the mature nervous system and the targets of most p38 inhibitor drug development^26^,^34^. To identify which p38 isoforms mediate BAA-induced prodynorphin expression, p38α and p38β expression in cultured primary microglia was specifically silenced using the RNA interference technology. Compared to the nonspecific oligonucleotide control, transfection with siRNA/p38α (0.4 μM) for 5 hours significantly reduced p38α mRNA expression by 62%, measured by qRT-PCR (*P* < 0.05 using one-way ANOVA followed by a post-hoc Student-Newman-Keuls test), whereas it did not significantly affect p38β mRNA expression (Fig. 3A). Western blot also showed the identical data, i.e., compared to the nonspecific oligonucleotide control, siRNA/p38α significantly reduced levels of p38α protein by 66% (*P* < 0.05 using one-way ANOVA followed by a post-hoc Student-Newman-Keuls test), but not p38β protein levels (Fig. 3B). Furthermore, transfection with 0.4 μM siRNA/p38β significantly reduced p38β mRNA by 64% and p38β protein by 55%, compared to the nonspecific oligonucleotide control (*P* < 0.05 using one-way ANOVA followed by a post-hoc Student-Newman-Keuls test), but did not affect expression of p38α mRNA or protein (Fig. 3C,D).

Treatment with 1 μM BAA for 1 hour significantly induced p38 phosphorylation by 2.3 fold. Pretreatment with siRNA/p38β (but not siRNA/p38α) completely inhibited BAA-induced p38 phosphorylation (*P* < 0.05 using two-way ANOVA followed by the post-hoc Student-Newman-Keuls test; Fig. 3E). However, pretreatment with siRNA/p38β (but not siRNA/p38α) completely suppressed BAA-stimulated prodynorphin expression (*P* < 0.05 using two-way ANOVA followed by the post-hoc Student-Newman-Keuls test; Fig. 3F).

**Figure 4:**
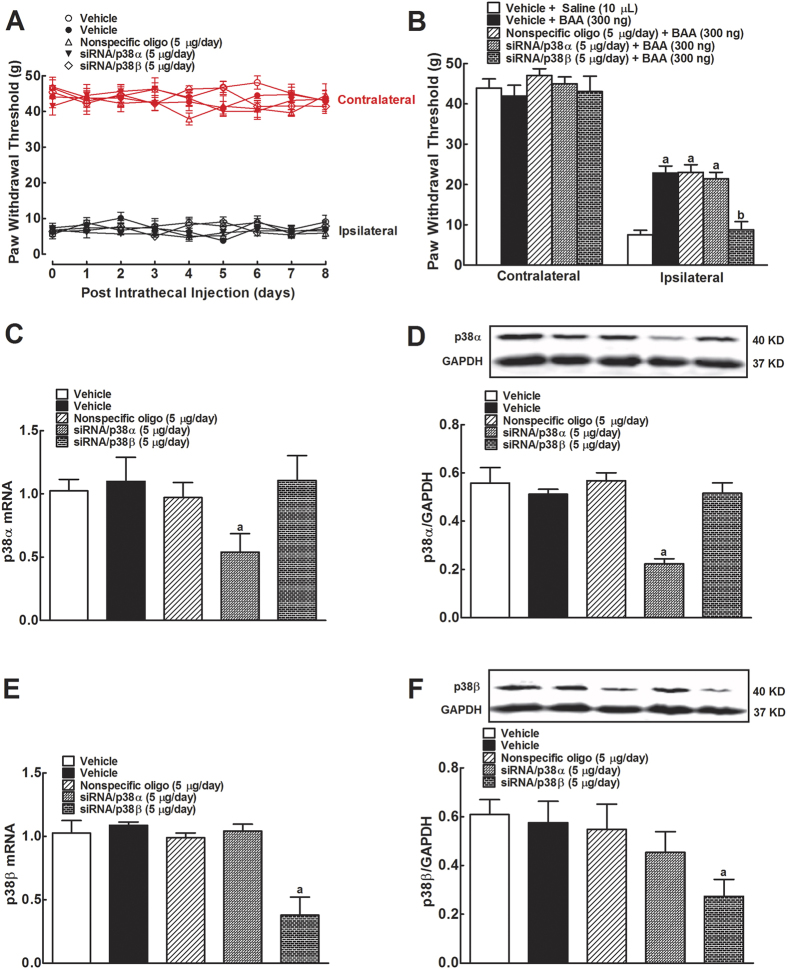
Differential effects of multiple-daily intrathecal injection of siRNA/p38α and siRNA/p38β MAPK on baseline pain thresholds (**A**), bulleyaconitine A (BAA)-induced mechanical antiallodynia (**B**), and expression of spinal p38α (**C** and **D**) and p38β (**E** and **F**) mRNA and protein in neuropathic rats. Neuropathic rats, induced by tight ligation of L5/L6 spinal nerves, and then received multiple-daily intrathecal injection of the vehicle (DOTAP, 30 μg/day), nonspecific oligos (5 μg/day), siRNA/p38α (5 μg/day) or siRNA/p38β (5 μg/day) for 7 days. The paw withdrawal thresholds were measured once a day using electronic von frey filaments. On the eighth day, a single bolus of saline (10 μL) or BAA (300 ng) was intrathecally injected and mechanical nociceptive behaviors were quantified 1 hour after injection. Spinal lumbar enlargements were immediately obtained after completion of behavioral assessments. Expressions of the mRNA of p38α, p38β and prodynorphin and protein of p38α and p38β referred to *gapdh* were determined using qRT-PCR and Western blot, respectively. The representative blots are shown in the upper panels of each panel. Data are expressed as means ± SEM (n = 6 in each group). ^a,b^*P* < 0.05 using one-way or two-way repeated measures ANOVA followed by the post-hoc student-Newman-Keuls test, compared with the control and BAA groups, respectively.

Further studies were undertaken to identify which isoform of p38 mediated BAA-induced spinal mechanical antiallodynia in neuropathy. Five groups of neuropathic rats received multiple daily intrathecal injections of the vehicle (Dotap, 40 μg/day), nonspecific siRNA oligonucleotide (5 μg/day), siRNA/p38α (5 μg/day), or siRNA/p38β (5 μg/day) for 7 days. The mechanical thresholds in both contralateral and ipsilateral paw were measured each morning before injection. On the eighth day, the rats received a single intrathecal injection of saline (10 μL for the first vehicle group) or BAA (300 ng for the remaining four groups). Mechanical thresholds were measured once 1 hour after BAA injection. As shown in Fig. 4A, consecutive daily intrathecal injections of siRNA/p38α and siRNA/p38β over 7 days did not significantly alter mechanical thresholds in either contralateral or ipsilateral paws, compared with the vehicle or nonspecific siRNA oligonucleotides control groups. On the 8th day, single intrathecal injection of BAA (300 ng) produced marked mechanical antiallodynia in the ipsilateral paws. Knockdown of p38β gene using siRNA/p38β completely prevented BAA mechanical antiallodynia (*P* < 0.05 using two-way ANOVA followed by the post-hoc Student-Newman-Keuls test), whereas multiple daily injections of siRNA/p38α was not able to block BAA-induced mechanical antiallodynia (Fig. 4B).

After completion of the behavior test, all rats were immediately sacrificed and spinal lumbar segments were obtained for later gene and protein assessment. Compared to the nonspecific siRNA oligonucleotides control, multiple daily intrathecal injections of siRNA/p38α significantly reduced p38α mRNA level by 51% (*P* < 0.05 using one-way ANOVA followed by post-hoc Student-Newman-Keuls test), but not p38β mRNA (Fig. 4C). Western blot showed similar data, i.e., siRNA/p38α significantly reduced p38α protein expression by 61% (*P* < 0.05 using one-way ANOVA followed by post-hoc Student-Newman-Keuls test), but not p38β protein (Fig. 4D). In contrast, multiple daily intrathecal injections of siRNA/p38β significantly reduced p38β mRNA level by 62% and protein by 50% (*P* < 0.05 using one-way ANOVA followed by post-hoc Student-Newman-Keuls test), but not p38α mRNA or protein (Fig. 4E,F).

### The cAMP-PKA signaling mediates BAA-induced prodynorphin expression in primary microglia

**Figure 5:**
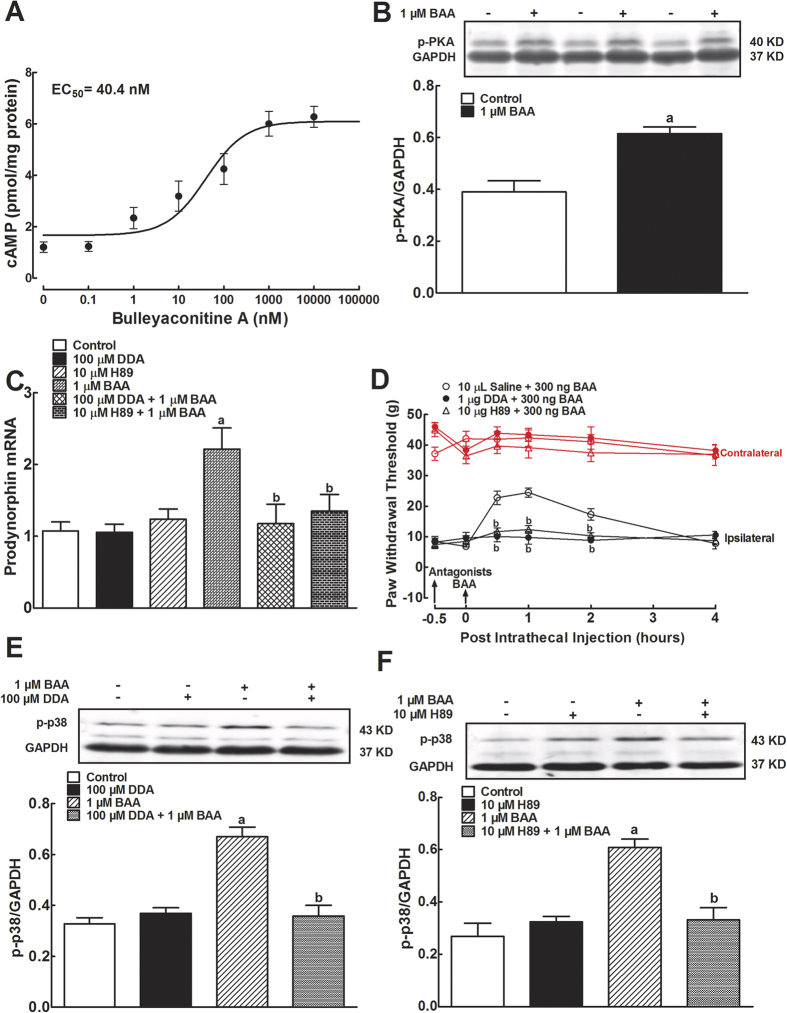
Stimulatory effects of bulleyaconitine A (BAA) on the intracellular cAMP level (**A**) and PKA phosphorylation (**B**), and blockade effects of the specific adenylate cyclase inhibitor DDA and PKA inhibitor H-89 on BAA-stimulated prodynorphin gene expression (**C**) in primary cultures of microglia, and spinal mechanical antiallodynia in neuropathic rats (**D**). Primary microglial cells were collected from the spinal cord of 1-day-old neonatal rats. For the cAMP measurement, these microglia were incubated with 1 μM BAA for 30 minutes and the intracellular cAMP accumulation was assayed using a commercial ELISA kit. For phosphorylation of PKA and p38, 1 μM BAA was incubated with cultured microglia for 1 hour, and subjected to Western blot analysis. The representative blots are shown in the upper panels of each panel. For gene expression, the MAPK activation inhibitors were incubated 30 minutes before BAA treatment, and primary microglial cells were collected 2 hours later. The level of dynorphin A precursor prodynorphin mRNA was determined using qRT-PCR and normalized to *GAPDH* mRNA. For mechanical antiallodynia, neuropathic rats were induced by tight ligation of L5/L6 spinal nerves, and mechanical thresholds were measured in both contralateral and ipsilateral paws. Blockade effects of DDA (**E**) and H-89 (**F**) on BAA-induced p38 phosphorylation were also assessed. Data are presented as means ± SEM (n = 3 in each treatment with two independent repeats in cultured primary microglial cells, and n = 6 in each group of neuropathic rats). ^a,b^*P* < 0.05 using the unpaired and two-tailed Student’s *t*-test or two-way repeated measures ANOVA followed by the post-hoc student-Newman-Keuls test, compared with the control and BAA groups, respectively.

Primary microglial cells were grown and treated with different BAA concentrations (10^−10^, 10^−9^, 10^−8^, 10^−7^, 10^−6^, and 10^−5^ M) for measurement of intracellular cAMP levels using a commercial ELISA kit 30 minutes later. Compared to the control, BAA treatment significantly enhanced the intracellular cAMP level in a concentration-dependent manner, with an EC_50_ of 40.4 nM (Fig. 5A). BAA-stimulated PKA activation was then determined with Western blot analysis. As shown in Fig. 5B, 1 μM BAA treatment of cultured primary microglia for 1 hour significantly increased PKA phosphorylation by 58% (*P* < 0.05 using unpaired and two-tailed Student t-test).

To assess whether BAA-induced prodynorphin expression and mechanical antiallodynia were through the cAMP/PKA signaling, the specific adenylate cyclase inhibitor DDA and PKA activation inhibitor H-89 were employed in cultured primary microglia and neuropathic rats. Treatment with 1 μM BAA for 2 hours significantly increased expression of prodynorphin mRNA by 2.1 fold, whereas DDA (100 μM) and H-89 (10 μM)^35^,^36^ did not significantly affect the basal prodynorphin expression. Pretreatment (1 hour before) with DDA or H-89 completely prevented BAA-increased prodynorphin expression (*P* < 0.05 by two-way ANOVA followed by the post-hoc Student-Newman- Keuls test; Fig. 5C).

Pretreatment of neuropathic rats with DDA (1 μg) and H-89 (10 μg)^37^ did not significantly affect baseline paw withdrawal thresholds in response to mechanical stimulation in both contralateral or ipsilateral paws, but completely prevented BAA-induced mechanical antiallodynia (*P* < 0.05 using two-way ANOVA followed by a post-hoc Student-Newman-Keuls test; Fig. 5D).

To further determine whether BAA-induced p38 phosphorylation was cAMP/PKA-dependent, DDA and H-89 were applied in cultured primary microglia. We found that pretreatment (1 hour before) with DDA (100 μM) and H-89 (10 μM) did not significantly affect the basal p38 phosphorylation level, but completely blocked BAA-upregulated p38 phosphorylation (*P* < 0.05 using two-way ANOVA followed by the post-hoc Student-Newman-Keuls test; Fig. 5E,F).

### A GsPCR possibly mediates BAA-induced prodynorphin expression in primary microglia

**Figure 6:**
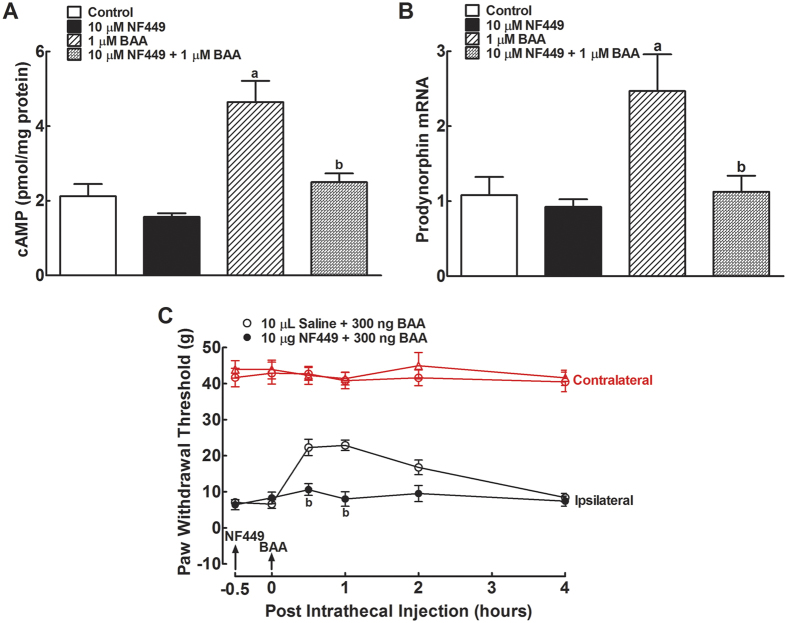
Blockade effects of the Gs-protein inhibitor NF449 on bulleyaconitine (BAA)-stimulated cAMP accumulation (**A**) and prodynorphin mRNA expression (**B**) in primary cultures of microglia, and spinal mechanical antiallodynia in neuropathic rats (**C**). Primary microglial cells were collected from the spinal cord of 1-day-old neonatal rats. For the cAMP measurement, microglial cells were incubated with 1 μM BAA for 30 minutes and subjected to ELISA analysis of the intracellular cAMP level using a commercial ELISA kit. For gene expression, the cultured cells were collected 2 hours after incubation with test reagents and subjected to qRT-PCR analysis of the dynorphin A precursor prodynorphin mRNA referred to GAPDH mRNA. For mechanical antiallodynia, neuropathic rats were induced by tight ligation of L5/L6 spinal nerves, and mechanical thresholds were measured in both contralateral and ipsilateral paws. Data are presented as means ± SEM (n = 3 in each treatment with two independent repeats in cultured primary microglial cells, n = 6 in each group of neuropathic rats). ^a,b^*P* < 0.05 using two-way repeated measures ANOVA followed by the post-hoc student-Newman-Keuls test, compared with the control and BAA groups, respectively.

To explore Gs-protein involvement in BAA-mediated cAMP production and subsequent prodynorphin expression, we employed a Gs inhibitor NF449 in cultured microglia. NF449 was identified as a selective inhibitor of the Gs signaling, with limited effects on the prototypical Gi/Go and Gq signaling^38^. BAA treatment (1 μM) for 30 minute significantly increased intracellular cAMP level, whereas NF449 treatment (10 μM)^39^ in cultured primary microglial cells did not significantly affect the basal intracellular cAMP level. However, 30 minutes NF449 pretreatment completely inhibited BAA-increased cAMP accumulation (*P* < 0.05 using two-way ANOVA followed by a post-hoc Student-Newman-Keuls test; Fig. 6A).

NF449 treatment (10 μM) did not significantly affect basal prodynorphin gene expression but BAA (1 μM) markedly increased prodynorphin expression. 30 minutes NF449 pretreatment completely suppressed BAA-stimulated prodynorphin mRNA level (*P* < 0.05 using two-way ANOVA followed by a post-hoc Student-Newman-Keuls test; Fig. 6B).

Furthermore, intrathecal BAA injection (300 ng) produced a marked mechanical antiallodynia in the ipsilateral paws in a time-dependent manner. 30 minutes pretreatment with intrathecal NF449 (10 μg)^40^,^41^ did not significantly change baseline paw withdrawal thresholds in either contralateral or ipsilateral paws, but completely prevented spinal BAA mechanical antiallodynia in the ipsilateral paws (*P* < 0.05 using two-way ANOVA followed by the post-hoc Student-Newman-Keuls test; Fig. 6C).

**Figure 7:**
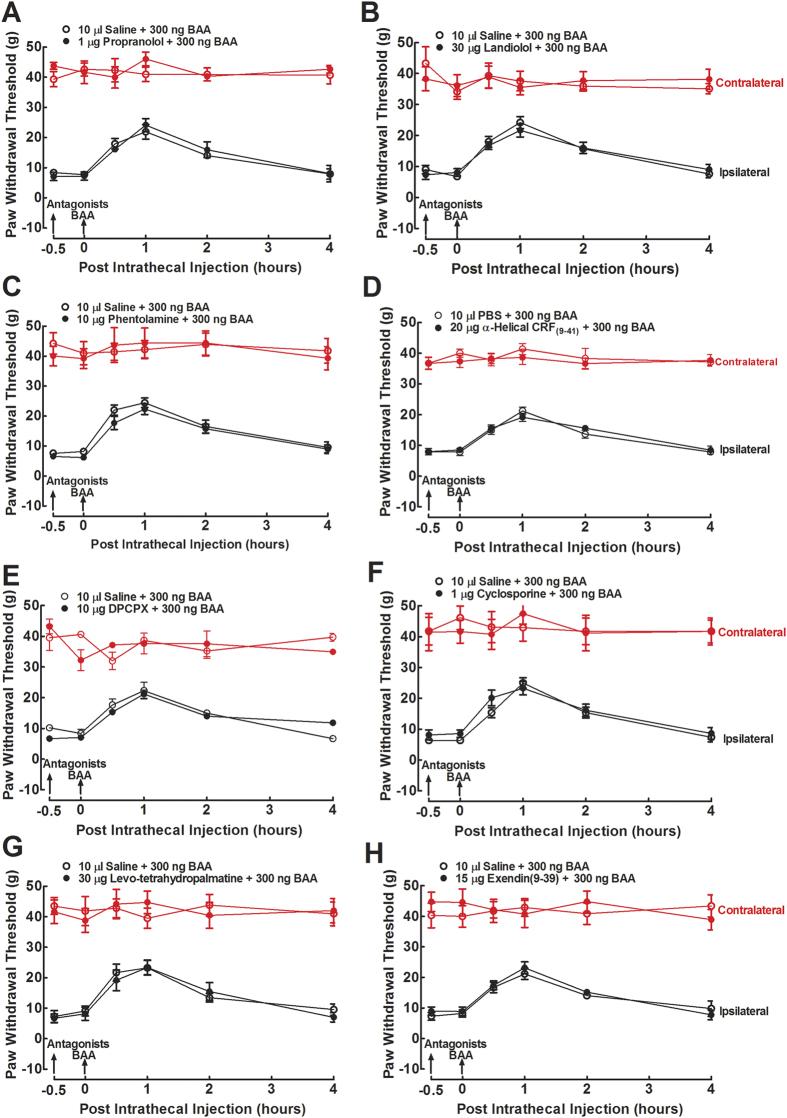
Ineffectiveness of the specific antagonists of the noradrenergic β-adrenoceptor, β_1_-adrenoceptor, α-adrenoceptor, corticotrophin-releasing factor receptor, adenosine A1 receptor, formyl peptide receptor, D1/D2 dopamine receptor and glucagon-like peptide-1 (GLP-1) receptor on bulleyaconitine (BAA)-induced spinal mechanical antiallodynia in neuropathic rats. Neuropathic rats, induced by tight ligation of L5/L6 spinal nerves, received intrathecal injection of saline or propranolol, landiolol, phentolamine, α-helical CRF(9–41), DPCPX, cyclosporine, levo-tetrahydropalmatine or exendin(9–39) followed by an intrathecal injection of BAA 30 minutes later. Mechanical withdrawal thresholds in both contralateral and ipsilateral paws were measured before and 0.5, 1, 2 and 4 hours after the second injection. Data are presented as means ± SEM (n = 3 in each group).

Lastly, as BAA-induced mechanical antiallodynia in neuropathy is clearly an *in vivo* indicator of spinal microglial prodynorphin expression and dynorphin A release^9^,^10^,^11^,^42^, 8 specific GPCR antagonists were intrathecally employed in neuropathic rats to identify the proposed GsPCR responsible for BAA-induced dynorphin A expression. Each two groups of neuropathic rats (n = 3 in each group) received intrathecal injections of saline (10 μL) or different GPCR antagonists followed by an intrathecal BAA injection (300 ng) 30 minutes later. These antagonists specifically targeted the following GPCRs, i.e., the noradrenergic β-adrenoceptor (propranolol, 1 μg)^43^, β_1_-adrenoceptor (landiolol, 30 μg)^44^, α-adrenoceptor (phentolamine, 10 μg)^45^,^46^, corticotrophin-releasing factor receptor (α-helical CRF(9–41), 20 μg)^47^, adenosine A1 receptor (DPCPX, 10 μg)^48^, formyl peptide receptor (cyclosporine, 1 μg)^49^, D1/D2 dopamine receptor (levo-tetrahydropalmatine, 30 μg)^50^, and glucagon-like peptide-1 (GLP-1) receptor (exendin(9–39), 15 μg)^51^. Mechanical withdrawal thresholds in both contralateral and ipsilateral paws were measured before and 0.5, 1, 2 and 4 hours after the second injection. As shown in Fig. 7, intrathecal BAA injection produced a time-dependent mechanical antiallodynia. However, treatment with propranolol (Fig. 7A), landiolol (Fig. 7B), phentolamine (Fig. 7C), α-helical CRF(9–41) (Fig. 7D), DPCPX (Fig. 7E), cyclosporine (Fig. F), levo-tetrahydropalmatine (Fig. 7G), or exendin(9–39) (Fig. 7H) failed to significantly reduce BAA-induced mechanical antiallodynia, although they are known to sufficiently block their respective spinal GPCR activities.

## Discussion

**Figure 8:**
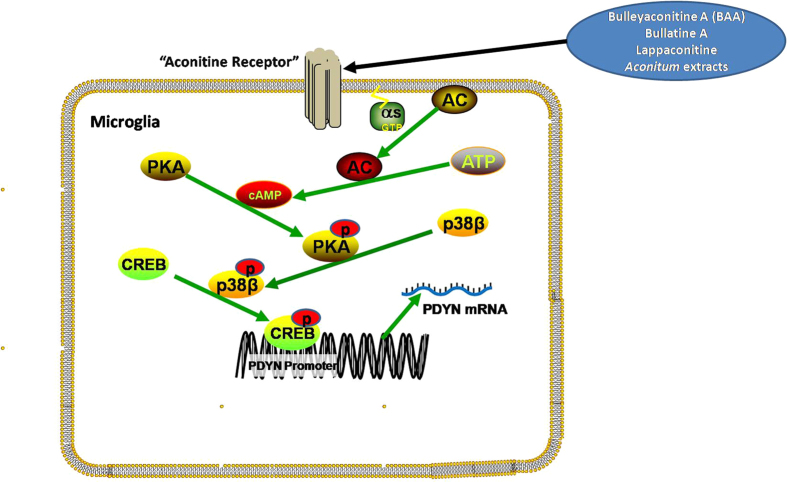
Illustration of the proposed signal transduction pathway of bulleyaconitine A (BAA), bullatine A, lappaconitine, and the *Aconitum* extracts-induced prodynorphin expression in microglia. Following activation of the “aconitine receptor”, the Gs-protein, cAMP/PKA, p38β (but not ERK, JNK or p38α) MAPK and CREB (but not NF-κB) signals are successively activated, which mediates prodynorphin expression.

In the current study, we have dissected and proposed signal transduction pathways for aconitines (represented by BAA) to stimulate prodynorphin expression in microglia. By binding to an unclarified GsPCR - “aconitine receptor” which is possibly specifically localized on microglial cell membrane, BAA, other aconitines (such as aconitine, bullatine A and lappaconitine) and the *Aconitum* extracts are able to activate Gs-protein and in turn trigger adenylate cyclase and produce a large amount of cAMP, which further stimulates PKA and subsequent p38β phosphorylation. The *p*-p38β then translocates to the nucleus and activates the transcription factor CREB, leading to an increase in prodynorphin transcription. The proposed signal transduction pathway is presented in Fig. 8. The illustration of the entire signal transduction pathway of aconitines further supports the notion that their antinociception is through spinal microglial prodynorphin expression and dynorphin A release, but not through the interactions with voltage-gated sodium channels which is not involved in the Gs-PKA-p38-CREB pathway. Furthermore, our results also highlight the selective role of p38β in pain transduction and provide an opportunity to identify the unclarified GsPCR, i.e., “aconitine receptor”.

CREB, a member of the basic leucine zipper family of the transcription factors, regulates a large and diverse group of gene transcription and function in cellular responses to various physiological stimuli, including neurotransmitters, growth factors, and cell stress^20^. Specifically, the transcriptional activity induced by phospho-CREB initiates binding to CRE present in the target gene promoter^52^. Previous studies showed that CREB regulated the transcription of prodynorphin gene in neurons^22^,^23^, while our current study demonstrated that BAA induced a robust CREB phosphorylation and prodynorphin expression in primarily cultured microglia, which was completely inhibited by the selective CREB inhibitor KG-501, indicating that BAA-stimulated microglial prodynorphin expression was mediated by CREB activation. In contrast, BAA did not induce phosphorylation of NF-κB, which is also a pleiotropic factor to promote transcription of a wide variety of genes. In microglia, activated NF-κB translocates into the nucleus and regulates the synthesis and release of proinflammatory cytokines to play a pivotal role in neuroinflammation^53^. However, bullatine A and lappaconitine did not significantly inhibit microglial expression of proinflammatory cytokines, including tumor necrosis factor (TNF)-α, interleukin (IL)-6, and IL-1β^11^,^12^. Both results rule out the possible involvement of NF-κB in mediation of BAA signaling transduction in microglia.

CREB is initially identified as a PKA substrate and phosphorylation of the serine-133 site makes it function as a stimulus-dependent transcriptional activator^21^. Another alternative pathway of the p38/CREB is also observed later, i.e., CREB phosphorylation can also be directly induced by p38 MAPK at the same serine 133 site^22^. Thus, these classic and alternative pathways do exist in different cells and respond to different gene signaling^54^,^55^. In our study, we found that selective p38 activation inhibitor SB203580 was able to entirely abolish BAA-activated microglial CREB phosphorylation and prodynorphin expression, suggesting that p38 MAPK activation plays an obligatory intermediate role in PKA-induced CREB’s regulation of prodynorphin expression in microglia.

We demonstrated that BAA specifically activated p38 without a significant effect on ERK or JNK activation. The selective p38 inhibitor but not the ERK or JNK inhibitor could entirely eliminate BAA-increased prodynorphin expression in cultured primary microglia. Our current data were supported by recent studies showing that aconitine treatment of myocardial cells induced p38 phosphorylation and cell apoptosis, both of which were significantly attenuated by SB203580 treatment^56^, and that the microglial inhibitor minocycline completely inhibited aconitine-, BAA- and bullatine A-increased dynorphin A gene and protein expression and antinociception in neuropathy^9^,^10^,^11^,^42^. Minocycline has been shown to inhibit spinal p38 activation^57^,^58^, particularly in a neuropathic pain condition^59^, although it was also shown to inhibit ERK^60^ and JNK activity^61^. Taken altogether, our data revealed that BAA specifically activated p38 and subsequent CREB phosphorylation leading to prodynorphin expression. Furthermore, our recent study showed that GLP-1 receptor agonism-induced β-endorphin expression was solely mediated by a p38-dependent CREB activation^62^. These data are of significance in pain transmission and transduction, as activation of p38 is an important intracellular signaling event which exclusively occurred in microglia and in turn regulated pain hypersensitivity, whereas activation of ERK and JNK activation usually occurs in dorsal horn neurons or astrocytes (but not in microglia) after spinal nerve injury^63^. Furthermore, p38 is a downstream target of several signal molecules^27^ and activation of cAMP/PKA to phosphorylation of MAPKs has been shown to be an important signal transduction process. We demonstrated that BAA-induced phosphorylation of p38 and prodynorphin expression were completely suppressed by DDA and H-89, highlighting that BAA-induced p38 phosphorylation is cAMP/PKA-dependent in microglia.

However, our finding seems contradictory as it is known that p38 phosphorylation was also involved in expression of pro-inflammatory cytokines, IL-1β, IL-6, and TNF-α^64^,^65^. It is true that p38 has α, β, γ, and δ isoforms and that α and β are the major isoforms in the mature nervous system^26^,^34^,^66^. The p38 α isoform was responsible for expression of pro-inflammatory cytokines^67^,^68^,^69^,^70^. While p38β appeared to be the most abundant isoform in spinal microglia^34^, it was not involved in inflammation and tissue damage^69^,^70^. We recently evidenced that GLP-1 receptor agonism-induced β-endorphin expression and mechanical antiallodynia in neuropathy were fully mediated by p38β activation, whereas lipopolysaccharides-induced expression of proinflammatory cytokines was partially mediated by p38α activation^62^. Our data further confirmed that p38β activation mediates BAA-induced prodynorphin expression in cultured primary microglial cells.

Many investigators have demonstrated that nerve injury would induce p38 phosphorylation in spinal cord microglia, which contributed to development and maintenance of neuropathic pain^26^. For instance, p38 activation in the neuropathic pain state exclusively occurred in microglia, but not in neurons or astrocytes in the dorsal horn^71^. However, there are contradictory findings regarding p38 as a target molecule for treatment of chronic pain in experimental animals, especially in established neuropathic pain. It was reported that selective p38 activation inhibitor SB203580 cannot reverse neuropathic pain when given to animals after neuropathy was established^72^. Our recent study reported that knockdown of spinal p38α or p38β expression was not antiallodynic^62^. Our current results demonstrated that multiple-daily injections of siRNA/p38α or siRNA/p38β did not alter mechanical thresholds in the ipsilateral paws in neuropathic rats. All these results together suggest that spinal p38 or its α or β isoform does not regulate basal pain states.

Our study revealed that BAA treatment dose-dependently enhanced cAMP accumulation and PKA phosphorylation in cultured primary microglial cells. BAA-induced prodynorphin expression in microglia were completely blocked by DDA and H-89, and particularly the specific Gs-protein inhibitor NF449, suggesting that BAA-increased prodynorphin expression was mediated by the Gs/cAMP/PKA signaling pathway. Thus, we speculate that a GsPCR expressed on microglial cell membrane could be the target molecule for BAA to stimulate prodynorphin expression and dynorphin A release. We tentatively name the unidentified GsPCR as the “aconitine receptor”.

GPCRs are the largest family of receptors to respond for a variety of stimuli, ranging from neurotransmitters and hormones to light and mechanical pressure, and represent targets for approximately 40% of all approved drugs in the clinic. It would be extremely valuable to illustrate the chemical identity of the “aconitine receptor” on microglia. In an effort to identify the “aconitine receptor”, we assessed the blockade ability of several specific GPCR antagonists in neuropathy on BAA mechanical antiallodynia, which has been demonstrated to be induced by spinal microglial dynorphin A expression (Li *et al*., 2016a; Li *et al*., 2016b; Huang *et al*.^11^; Huang *et al*.^42^). The GPCR receptors assessed in our study included the β-adrenoceptor (and β_1_-adrenoceptor), α-adrenoceptor, corticotrophin-releasing factor receptor, adenosine A1 receptor, formyl peptide receptor, D1/D2 dopamine receptor, and GLP-1 receptor. However, all the antagonists failed to alter BAA mechanical antiallodynia in neuropathy. It is worth noting that the D1/D2 dopamine receptor antagonist levo-tetrahydropalmatine did not alter BAA-induced mechanical antiallodynia, suggesting that BAA-induced dynorphin A expression is not involved in activation of D1/D2 dopamine receptors in microglia, whereas activation of D1 dopamine receptor was reported to stimulate dynorphin A expression in a cAMP/PKA/CREB signaling-dependent manner in nucleus accumbens and striatonigral neurons (Carlezon *et al*.^23^; Muschamp and Carlezon, 2015). Further studies are warranted to utilize techniques, such as GsPCR binding or functional screening assays of large scale and chemical proteomics, to identify the particular GsPCR involved in BAA-induced spinal microglial dynorphin A expression and mechanical antiallodynia in animals.

## Materials and Methods

### Drugs and reagents

BAA was purchased from Zelang Bio-Pharmaceuticals (Nanjing, China) and NF449 was from Tocris Bioscience (Bristol, UK). 2′,5′-dideoxyadenosine (DDA) and N-(2-(p-bromocinnamylamino)ethyl)-5-isoquinolinesulfonamide (H-89) were obtained from Santa Cruz Biotechnology (Santa Cruz, CA, USA), while KG-501 and SP600125 were from Sigma-Aldrich (St. Louis, MO, USA) and SB203580 and U0126 were from Selleck Chemicals (Houston, TX, USA). Levo-tetrahydropalmatine was a gift from Dr. Yan Zhang at Shanghai Jiao University School of Pharmacy. KG-501, SB203580, U0126, and SP600125 were dissolved in dimethyl sulfoxide (DMSO) and made 20% stock solution in saline, while other drugs and reagents were dissolved in 0.9% normal saline.

### siRNA and transfection

The small interference of double-stranded RNA (siRNA) targeting the cDNA sequence of the rat p38α (siRNA/p38α), p38β (siRNA/p38β), and the nonspecific oligonucleotides were designed and synthesized by Shanghai GenePharma (Shanghai, China). Their sequences were siRNA/p38α, 5′-GCACGAGAAUGUGAUUGGUTT-3′ and 5′-ACCAAUCACAUUCUCGUGCTT-3′; siRNA/p38β, 5′-GCACGAGAACGUCAUAGGATT-3′ and 5′-UCCUAUGACGUUCUCGUGCTT-3′; nonspecific oligonucleotides, 5′-UUCUCCGAACGUGUCACGUTT-3′ and 5′-ACGUGACACGUUCGGAGAATT-3′. To formulate siRNAs, 1,2-dioleoyloxy-3-trimethylammonium-propane (DOTAP, Sigma-Aldrich) was dissolved in water (5 mg/mL, pH 7.0). For the *in vitro* transfection, 50 μL diluted siRNA solution was added into 50 μL diluted DOTAP solution in a DOTAP:RNA mass ratio of 1:8, thoroughly mixed, and then incubated at the room temperature for 15 minutes.

Primary microglia were seeded into 6 or 24-well plates and grown overnight. The siRNA-DOTAP complex was then added into the plates with 300 μL/500 μL basic Dulbecco’s modified Eagle’s medium (DMEM) to make the final siRNA concentration of 5 μg/mL. The plates were then incubated in a humidified incubator with 5% CO_2_ at 37 °C for 5 hours. Cells were further cultured for 24 hours after refreshing with the cultural medium. For the *in vivo* experiments, the siRNA-DOTAP complex was formulated in the same DOTAP:RNA mass ratio of 1:8 and intrathecally injected into rats for successive 7 days.

### Experimental animals

Male adult (200 ± 20 g body weight) and 1-day-old Wistar neonatal rats were purchased from the Shanghai Experimental Animal Institute for Biological Sciences (Shanghai, China). The adult rats were maintained four per cage, received food and water *ad libitum*, and housed in the Shanghai Jiao Tong University Experimental Animal Center (Shanghai, China) with thick sawdust bedding at standard room temperature (22 ± 2 °C), under conditions of a 12/12-hr reversed light-dark cycle (7:00 am–7:00 pm). The adult rats were accustomed to the laboratory environment for 3–5 days before our experiments. Experimental groups (n = 6 in each group except for the target screening study as indicated in the text) were randomly assigned, and the investigator was blinded for the behavior tests. This study was approved by the Animal Care and Welfare Committee of Shanghai Jiao Tong University and carried out in accordance with the animal care guidelines of the US National Institutes of Health.

### Primary culture of microglial cells

Microglial cells were isolated from the spinal dorsal horn of 1-day-old neonatal rats. The isolated spinal cords were minced and then incubated with 0.05% trypsin solution. Dissociated cells were suspended in DMEM supplemented with 10% (vol/vol) fetal calf serum and penicillin (100 U/mL) and streptomycin (100 μg/mL) and plated in 75-cm^2^ tissue culture flasks (1 × 10^7^ cells/flask) that were pre-coated with poly-L-lysine and maintained in a 5% carbon monoxide incubator at 37 °C. After culture for 8 days, microglial cells were prepared as floating cell suspensions by shaking the flasks at 260 rpm for 2 hours. The aliquots were transferred to plates and unattached cells were removed by washing with serum-free DMEM. Harvested microglial cells showed a purity >95%, as determined by the OX42 immunoreactivity (Gong *et al*., 2014).

### RNA isolation and quantitative reverse transcription-polymerase chain reaction (qRT-PCR)

The spinal dorsal lumbar enlargements were collected and homogenized using an electronic microhomogenizer at 4,000 rpm for 10 seconds in TRIzol (Invitrogen, Carlsbad, CA, USA) on ice, while total RNA from cultured microglial cells was also isolated directly using TRIzol. The RNA samples of 1 μg each were reversely transcribed into cDNA using a ReverTra Ace qPCR RT-Kit (Toyobo Co., Osaka, Japan). qPCR was then carried out in a Mastercycler ep realplex (Eppendorf, Germany) using Realmaster Mix (SYBR Green I) (Toyobo Co.) according to a previous study^73^. The fold change was calculated using the 2^−ΔΔCt^ method after normalization to *GAPDH*. The primers were *GAPDH* (5′-CCA AGG TCA TCC ATG ACA AC-3′ and 5′-TCC ACA GTC TTC TGA GTG GC-3′)^51^ and *PDYN* (5′-ACT GCC TGT CCT TGT GTT CC-3′ and 5′-CCA AAG CAA CCT CAT TCT CC-3′)^74^.

### Protein extraction and Western blot

Cultured microglia were seeded into 6-well plates at a density of 5 × 10^6^ cells per well and grown overnight and then harvested and lysed in the radioimmunoprecipitation assay (RIPA) lysis buffer with addition of the phosphates inhibitor cocktail and the protease inhibitor cocktail (Biotool, Houston, USA). Briefly, cells were scraped into the lysis buffer and homogenized by passing through a 21G needle and cell lysates were centrifuged at 13,000 g at 4 °C for 15 minutes. The supernatants were then collected and the protein concentration was quantified using a bicinchoninic acid (BCA) assay according to the manufacturer’s protocol (Beyotime Institute of Biotechnology, Jiangsu, China). The homogenates were diluted 1:1 (v/v) with 5× sodium dodecyl sulfate (SDS) sample buffer (Bio-Rad, Hercules, CA, USA) and boiled for 10 minutes at 95 °C in the SDS sample buffer. The protein samples were then separated in 12% sodium dodecyl sulfate-polyacrylamide gel electrophoresis (SDS-PAGE) gels and transferred onto a polyvinylidene fluoride (PVDF) membrane using the electrophoretic method. The membrane was then blocked in 5% skim milk powder in Tris-based saline-Tween 20 (TBST) at room temperature for one hour, and then incubated at 4 °C overnight with rabbit monoclonal primary antibodies raised against phospho-p38, phospho-ERK, phospho-JNK, p38α, p38β, phospho-CREB (Cell Signaling Technology, Danvers, MA, USA) at a dilution of 1:1,000, anti-phospho-PKA catalytic subunit (Santa Cruz Biotechnology) at a dilution of 1:200, and a mouse monoclonal antibody against GAPDH (Protein Tech Group, Chicago, USA) at a dilution of 1:4,000, respectively. The membranes were then washed four times in TBST (10 minutes each) and further incubated 1 hour with a secondary peroxidase conjugated goat anti-rabbit and goat anti-mouse antibody, and washed four times with TBST. Protein bands were visualized using the IRDye 800-conjugated affinity purified goat anti-rabbit IgG and IRDye 700-conjugated affinity purified goat anti-mouse IgG (Cell Signaling Technology, Danvers, MA, USA) in the Odyssey Infrared Imaging system (Li-Cor Biosciences, Lincoln, NE, USA). The band intensity was quantitated using a computer-assisted image analysis program (ImageJ Software, National Institutes of Health, Bethesda, MD, USA) and compared to the house-keeping protein, i.e., the band intensity ratios of phospho-p38/GAPDH, phospho-ERK/GAPDH, phospho-JNK/GAPDH, phospho-PKA/GAPDH, p38α/GAPDH, p38β/GAPDH, and phospho-CREB/GAPDH were obtained to quantify the respective target protein expression^51^,^75^.

### Intracellular cAMP assay

Cultured microglial cells (5 × 10^6^) were seeded into 6-well plates and treated with the phosphodiesterase inhibitor IBMX (Sigma-Aldrich) at 0.5 mM in serum-free medium for 30 minutes before treatment with different concentrations (10^−10^, 10^−9^, 10^−8^, 10^−7^, 10^−6^, and 10^−5^ M) of bulleyaconitine A. 30 minutes after incubation, microglial cells were lysed using the R&D Cell Lysis Buffer and cAMP release was determined using a commercial cAMP enzyme immunoassay kit (R&D Systems, Minneapolis, USA) according to the manufacturer’s protocols. The concentration of cAMP was calculated using a standard curve using the Assay Zap (Biosoft, Cambridge, UK) and normalized. Data represented two independent experiments with triplicate^76^.

### Intrathecal catheterization and injection in rats

A 18-cm polyethylene catheter (PE-10: 0.28 mm inner diameter and 0.61 mm outer diameter) (Clay Adams, Parsippany, NJ, USA) with a volume of 13 μL was inserted into the rat lumbar level of the spinal cord under inhaled isoflurane anesthesia (4% for induction and 1% for maintenance) run by an anesthesiameter (Ugo Basile Gas Anesthesia System, Comerio, Italy). Two days after recovery from anesthesia, the installation of the catheter in the spinal cord was verified by administering 4% lidocaine (10 μL followed by 15 μL of saline for flushing). Rats that did not induce motor impairment following insertion of the intrathecal catheter and developed immediate bilateral paralysis of the hindlimbs following intrathecal lidocaine were selected for the study. For the intrathecal delivery, 10 μL of the drug solution was administered in a 50-μL microinjector (Shanghai Anting Micro-Injector Factory, Shanghai, China) followed by 15 μL saline flush^77^.

### Rat neuropathic pain model and behavioral assessment of mechanical allodynia

The unilateral ligation of two spinal nerves was performed under inhaled isoflurane anesthesia run by the anesthesiameter. The left L5 and L6 spinal nerves were isolated and tightly ligated with 6–0 silk thread. After ligation, the wound was sutured and the rats were allowed to recover. As intrathecal injection was needed in this study, the intrathecal catheterization was performed in rats at the same time just before spinal nerve ligation. Of the spinal nerve-ligated animals, only those with marked unilateral allodynia to mechanical stimulation (hindlimb withdrawal thresholds in the operated side <8 g) and no major impairment were included in the study. Drug testing started 2–4 weeks after spinal nerve ligation surgery^78^,^79^).

To evaluate mechanical allodynia, the animals were acclimatized for at least 30 minutes to the test environment, namely a plexiglass box on a metal grid (0.5 × 0.5 cm). The hindpaw withdrawal threshold was measured by using a 2450 CE Electronic Von Frey hair (IITC Life Science Inc, Woodland Hill, CA, USA). The electronic hand-held transducer with a No. 15 monofilament (with forces ranging from 0.1 to 90 g) was applied perpendicularly to the medial surface of the hindpaws with the increasing force until the rat suddenly withdrew or licked the hindpaw. The lowest force to produced a withdrawal response was considered to be the threshold. Three repeated measurements were made at intervals of approximately 5 minutes and were averaged for each hindpaw at each time-point^78^,^79^.

### Statistical analysis

For the concentration–response curve analysis, the parameters, i.e., the minimum, maximum (E_max_), median effective concentration (EC_50_) and Hill coefficient (n) were calculated from individual concentration–response curves. To determine the parameters of the concentration–response curves, values of response (Y) were fitted by non-linear least-squares curves to the relation Y = a + bx, where x = (C)^n^/(EC_50_^n^ + (C)^n^), to give the value of EC_50_ and b (E_max_) yielding a minimum residual sum of squares of deviations from the theoretical curve^80^.

Data were presented as means ± standard error of the mean (SEM), and there were no missing data in this study. Statistical significance was evaluated using unpaired and two-tailed Student’s *t*-test or a one-way or two-way repeated measured analysis of variance (ANOVA) with the 5.01 Prism program (GraphPad Software Inc., San Diego, CA, USA). This was followed by a post-hoc Student-Newman-Keuls test when a statistically significant drug (dose) effect was observed (for the one-way ANOVA, the factor was drug (dose); for the two-way ANOVA, the factors were drug (dose), time, and their interaction). Probability values were two-tailed, and the statistical significant criterion P value was set to be 0.05.

## Additional Information

**How to cite this article:** Li, T.-F. *et al*. Molecular signaling underlying bulleyaconitine A (BAA)-induced microglial expression of prodynorphin. *Sci. Rep.* **7**, 45056; doi: 10.1038/srep45056 (2017).

**Publisher's note:** Springer Nature remains neutral with regard to jurisdictional claims in published maps and institutional affiliations.

## References

[b1] Isono T. . The analgesic mechanism of processed Aconiti tuber: the involvement of descending inhibitory system. Am J Chin Med 22, 83–94 (1994). 8030622 10.1142/S0192415X94000115

[b2] Suzuki Y. . Analgesic effect of benzoylmesaconine. Folia Pharmacol Japonica 102, 399–404 (1993). 10.1254/fpj.102.399 8282271

[b3] Suzuki Y., Goto K., Ishige A., Komatsu Y. & Kamei J. Antinociceptive effect of Gosha-jinki-gan, a Kampo medicine, in streptozotocin-induced diabetic mice. Jpn J Pharmacol 79, 169–175 (1999). 10202852 10.1254/jjp.79.169

[b4] Omiya Y., Goto K., Suzuki Y., Ishige A. & Komatsu Y. Analgesia-producing mechanism of processed Aconiti tuber: role of dynorphin, an endogenous kappa-opioid ligand, in the rodent spinal cord. Jpn J Pharmacology 79, 295–301 (1999). 10.1254/jjp.79.295 10230857

[b5] Xu H. . Pain-relieving effects of processed Aconiti tuber in CCI-neuropathic rats. J Ethnopharmacol 103, 392–397 (2006). 16183224 10.1016/j.jep.2005.08.050

[b6] Chodoeva A., Bosc J.-J. & Robert J. Aconitum alkaloids and biological activities, in Natural Products. Springer Berlin Heidelberg, 1503–1523 (2013).

[b7] Ameri A. The effects of Aconitum alkaloids on the central nervous system. Prog Neurobiol 56, 211–235 (1998). 9760702 10.1016/s0301-0082(98)00037-9

[b8] Tang X. C., Liu X. J., Lu W. H., Wang M. D. & Li A. L. Studies on the analgesic action and physical dependence of bulleyaconitine A. Acta Pharmaceut Sin 21, 886–891 (1986). 3591327

[b9] Li T.-F., Fan H. & Wang Y.-X. Aconitum-Derived Bulleyaconitine A Exhibits Antihypersensitivity Through Direct Stimulating Dynorphin A Expression in Spinal Microglia. J Pain 17, 530–548 (2016). 26762790 10.1016/j.jpain.2015.12.015

[b10] Li T.-F., Gong N. & Wang Y.-X. Ester hydrolysis differentially reduces aconitine-induced anti-hypersensitivity and acute neurotoxicity: Involvement of spinal microglial dynorphin expression and implications for Aconitum processing. Front. Pharmacol 7, 367 (2016). 27761113 10.3389/fphar.2016.00367 PMC5051147

[b11] Huang Q. . Bullatine A stimulates spinal microglial dynorphin A expression to produce anti-hypersensitivity in a variety of rat pain models. J Neuroinflammation 13, 214 (2016). 27577933 10.1186/s12974-016-0696-2 PMC5006272

[b12] Sun M.-L., Huang Q. & Wang Y.-X. The research on analgesic effect and mechanism of lappaconitine. Chin Pharmacol Bull 29, 81–81 (2015).

[b13] Chan T. Y. . Aconitine poisoning due to Chinese herbal medicines: a review. Veteri Human Toxicol 36, 452–455 (1994). 7839574

[b14] Ono T., Hayashida M., Tezuka A., Hayakawa H. & Ohno Y. Antagonistic effects of tetrodotoxin on aconitine-induced cardiac toxicity. J Nippon Med Sch 80, 350–361 (2013). 24189353 10.1272/jnms.80.350

[b15] Minokadeh A. . Cathepsin L participates in dynorphin production in brain cortex, illustrated by protease gene knockout and expression. Mol Cell Neurosci 43, 98–107 (2010). 19837164 10.1016/j.mcn.2009.10.001

[b16] Wahlert A., Funkelstein L., Fitzsimmons B., Yaksh T. & Hook V. Spinal astrocytes produce and secrete dynorphin neuropeptides. Neuropeptides 47, 109–115 (2013). 23290538 10.1016/j.npep.2012.10.006 PMC3606903

[b17] Dykstra L. A., Gmerek D. E., Winger G. & Woods J. H. Kappa opioids in rhesus monkeys. I. Diuresis, sedation, analgesia and discriminative stimulus effects. J Pharmacol Exp Ther 242, 413–420 (1987). 3612543

[b18] Conklin B. R. & Bourne H. R. Structural elements of Gα subunits that interact with Gβγ, receptors, and effectors. Cell 73, 631–641 (1993). 8388779 10.1016/0092-8674(93)90245-l

[b19] Knight B. L. & Fordham R. A. The separation, properties and possible subunit composition of adenosine 3′,5′-monophosphate-dependent protein kinases in brown adipose tissue. Biochim Biophys Acta 384, 102–111 (1975). 165829 10.1016/0005-2744(75)90099-6

[b20] Taylor S. S., Buechler J. A. & Yonemoto W. cAMP-dependent protein kinase: framework for a diverse family of regulatory enzymes. Annu Rev Biochem 59, 971–1005 (1990). 2165385 10.1146/annurev.bi.59.070190.004543

[b21] Frank D. A. & Greenberg M. E. CREB: a mediator of long-term memory from mollusks to mammals. Cell 79, 5–8 (1994). 7923377 10.1016/0092-8674(94)90394-8

[b22] Lonze B. E. & Ginty D. D. Function and regulation of CREB family transcription factors in the nervous system. Neuron 35, 605–623 (2002). 12194863 10.1016/s0896-6273(02)00828-0

[b23] Carlezon W. A. Jr., Duman R. S. & Nestler E. J. The many faces of CREB. Trends Neurosci 28, 436–445 (2005). 15982754 10.1016/j.tins.2005.06.005

[b24] Lucas T. F., Lazari M. F. M. & Porto C. S. Differential role of the estrogen receptors ESR1 and ESR2 on the regulation of proteins involved with proliferation and differentiation of Sertoli cells from 15-day-old rats. Mol Cellular Endocrinol 382, 84–96 (2014). 24056172 10.1016/j.mce.2013.09.015

[b25] Lee T.-M. & Chung T. Dipeptidyl peptidase IV inhibition attenuates ventricular vulnerability via cAMP/PKA/CREB pathway. Eur Heart J 34, P3308 (2013).

[b26] Ji R.-R. & Suter M. R. p38 MAPK, microglial signaling, and neuropathic pain. Mol Pain 3, 1 (2007). 17974036 10.1186/1744-8069-3-33 PMC2186318

[b27] Pyo H., Joe E., Jung S., Lee S. H. & Jou I. Gangliosides activate cultured rat brain microglia. J Biol Chem 274, 34584–34589 (1999). 10574921 10.1074/jbc.274.49.34584

[b28] Suzuki T. . Production and release of neuroprotective tumor necrosis factor by P2X7 receptor-activated microglia. J Neurosci 24, 1–7 (2004). 14715932 10.1523/JNEUROSCI.3792-03.2004 PMC6729576

[b29] Kim D. E., Kim B., Shin H. S., Kwon H. J. & Park E. S. The protective effect of hispidin against hydrogen peroxide-induced apoptosis in H9c2 cardiomyoblast cells through Akt/GSK-3beta and ERK1/2 signaling pathway. Exp Cell Res 327, 264–275 (2014). 25128810 10.1016/j.yexcr.2014.07.037

[b30] Han Z. N. . C-Jun N-terminal kinase is required for metalloproteinase expression, and joint destruction in inflammatory arthritis. J Clin Investigation 108, 73–81 (2001). 10.1172/JCI12466 PMC209341 11435459

[b31] Jin S. X., Zhuang Z. Y., Woolf C. J. & Ji R. R. p38 mitogen-activated protein kinase is activated after a spinal nerve ligation in spinal cord microglia and dorsal root ganglion neurons and contributes to the generation of neuropathic pain. J Neurosci 23, 4017–4022 (2003). 12764087 10.1523/JNEUROSCI.23-10-04017.2003 PMC6741086

[b32] Wang L. N. . Cancer-induced bone pain sequentially activates the ERK/MAPK pathway in different cell types in the rat spinal cord. Mol Pain 7, 48 (2011). 21722369 10.1186/1744-8069-7-48 PMC3150304

[b33] Zhuang Z. Y. . A peptide c-Jun N-terminal kinase (JNK) inhibitor blocks mechanical allodynia after spinal nerve ligation: respective roles of JNK activation in primary sensory neurons and spinal astrocytes for neuropathic pain development and maintenance. J Neurosci 26, 3551–3560 (2006). 16571763 10.1523/JNEUROSCI.5290-05.2006 PMC6673862

[b34] Svensson C. I. . Spinal p38beta isoform mediates tissue injury-induced hyperalgesia and spinal sensitization. J Neurochem 92, 1508–1520 (2005). 15748168 10.1111/j.1471-4159.2004.02996.x

[b35] Ballo A. W. . Dopamine modulates Ih in a motor axon. J Neurosci 30, 8425–8434 (2010). 20573890 10.1523/JNEUROSCI.0405-10.2010 PMC2908950

[b36] Saha A. . Prostaglandin E2 negatively regulates the production of inflammatory cytokines/chemokines and IL-17 in visceral leishmaniasis. J Immunol 193, 2330–2339 (2014). 25049356 10.4049/jimmunol.1400399

[b37] Lim G. . Expression of spinal NMDA receptor and PKCgamma after chronic morphine is regulated by spinal glucocorticoid receptor. J Neurosci 25, 11145–11154 (2005). 16319314 10.1523/JNEUROSCI.3768-05.2005 PMC6725649

[b38] Hohenegger M. . Gsalpha-selective G protein antagonists. Proc Natl Acad Sci USA 95, 346–351 (1998). 9419378 10.1073/pnas.95.1.346 PMC18220

[b39] Cheng Y. . Leucine deprivation stimulates fat loss via increasing CRH expression in the hypothalamus and activating the sympathetic nervous system. Mol Endocrinol 25, 1624–1635 (2011). 21719534 10.1210/me.2011-0028 PMC3165911

[b40] Ando R., Mehesz B., Gyires K., Illes P. & Sperlagh B. A comparative analysis of the activity of ligands acting at P2X and P2Y receptor subtypes in models of neuropathic, acute and inflammatory pain. Bri J Pharmacol 159, 1106–1117 (2010). 10.1111/j.1476-5381.2009.00596.x PMC2839268 20136836

[b41] Hohenegger M. . Gsα-selective G protein antagonists. Proceedings Nat Acad Sci 95, 346–351 (1998). 10.1073/pnas.95.1.346 PMC18220 9419378

[b42] Huang Q., Sun M. L., Chen Y., Li X. Y. & Wang Y. X. Concurrent bullatine A enhances morphine antinociception and inhibits morphine antinociceptive tolerance by indirect activation of spinal kappa-opioid receptors. J Ethnopharmacol 196, 151–159 (2017). 27989510 10.1016/j.jep.2016.12.027

[b43] Khasar S. G., Green P. G., Miao F. J. P. & Levine J. D. Vagal modulation of nociception is mediated by adrenomedullary epinephrine in the rat. Eur J Neurosci 17, 909–915 (2003). 12603283 10.1046/j.1460-9568.2003.02503.x

[b44] Wajima Z., Tsuchida H., Shiga T., Imanaga K. & Inoue T. Intravenous landiolol, a novel beta(1)-adrenergic blocker, reduces the minimum alveolar concentration of sevoflurane in women. J Clin Anesth 23, 292–296 (2011). 21663813 10.1016/j.jclinane.2010.11.001

[b45] Sherman S. E., Loomis C. W., Milne B. & Cervenko F. W. Intrathecal oxymetazoline produces analgesia via spinal alpha-adrenoceptors and potentiates spinal morphine. Eur J Pharmacol 148, 371–380 (1988). 2838306 10.1016/0014-2999(88)90115-x

[b46] Koo S. T., Lim K. S., Chung K., Ju H. & Chung J. M. Electroacupuncture-induced analgesia in a rat model of ankle sprain pain is mediated by spinal alpha-adrenoceptors. Pain 135, 11–19 (2008). 17537577 10.1016/j.pain.2007.04.034 PMC2268107

[b47] Stephens R. L. Jr., Yang H., Rivier J. & Tache Y. Intracisternal injection of CRF antagonist blocks surgical stress-induced inhibition of gastric secretion in the rat. Peptides 9, 1067–1070 (1988). 3266664 10.1016/0196-9781(88)90090-3

[b48] Lohse M. J. . 8-Cyclopentyl-1,3-dipropylxanthine (DPCPX)–a selective high affinity antagonist radioligand for A1 adenosine receptors. Naunyn-Schmiedeberg’s Archives Pharmacol 336, 204–210 (1987). 10.1007/BF00165806 2825043

[b49] Wenzel-Seifert K. & Seifert R. Cyclosporin H is a potent and selective formyl peptide receptor antagonist. Comparison with N-t-butoxycarbonyl-L-phenylalanyl-L-leucyl-L-phenylalanyl-L- leucyl-L-phenylalanine and cyclosporins A, B, C, D, and E. J Immunol 150, 4591–4599 (1993). 8387097

[b50] Mantsch J. R. . Levo-tetrahydropalmatine attenuates cocaine self-administration and cocaine-induced reinstatement in rats. Psychopharmacology 192, 581–591 (2007). 17361394 10.1007/s00213-007-0754-7

[b51] Gong N. . Activation of spinal glucagon-like peptide-1 receptors specifically suppresses pain hypersensitivity. J Neurosci 34, 5322–5334 (2014). 24719110 10.1523/JNEUROSCI.4703-13.2014 PMC6608999

[b52] Altarejos J. Y. & Montminy M. CREB and the CRTC co-activators: sensors for hormonal and metabolic signals. Nat Rev Mol Cell Biol 12, 141–151 (2011). 21346730 10.1038/nrm3072 PMC4324555

[b53] Makarov S. S. NF-kappaB as a therapeutic target in chronic inflammation: recent advances. Mol Med Today 6, 441–448 (2000). 11074370 10.1016/s1357-4310(00)01814-1

[b54] Nemeth Z. H. . Adenosine stimulates CREB activation in macrophages via a p38 MAPK-mediated mechanism. Biochem Biophys Res Com 312, 883–888 (2003). 14651954 10.1016/j.bbrc.2003.11.006

[b55] Wei H. . Mechanical antihypersensitivity effect induced by repeated spinal administrations of a TRPA1 antagonist or a gap junction decoupler in peripheral neuropathy. Pharmacol Biochem Behavior 150, 57–67 (2016). 10.1016/j.pbb.2016.09.007 27677209

[b56] Sun G. B. . Aconitine-induced Ca2+ overload causes arrhythmia and triggers apoptosis through p38 MAPK signaling pathway in rats. Toxicol Appl Pharmacol 279, 8–22 (2014). 24840785 10.1016/j.taap.2014.05.005

[b57] Hua X. Y. . Intrathecal minocycline attenuates peripheral inflammation-induced hyperalgesia by inhibiting p38 MAPK in spinal microglia. Eur J Neurosci 22, 2431–2440 (2005). 16307586 10.1111/j.1460-9568.2005.04451.x

[b58] Won K. A. . Participation of microglial p38 MAPK in formalin-induced temporomandibular joint nociception in rats. J Orofacial Pain 26, 132–141 (2012). 22558613

[b59] Hains B. C. & Waxman S. G. Activated microglia contribute to the maintenance of chronic pain after spinal cord injury. J Neurosci 26, 4308–4317 (2006). 16624951 10.1523/JNEUROSCI.0003-06.2006 PMC6674010

[b60] Cho I. H. . Minocycline markedly reduces acute visceral nociception via inhibiting neuronal ERK phosphorylation. Mol Pain 8, 13 (2012). 22364340 10.1186/1744-8069-8-13 PMC3342906

[b61] Song X. . Minocycline protects melanocytes against H2O2-induced cell death via JNK and p38 MAPK pathways. InterJ Mol Med 22, 9–16 (2008). 18575770

[b62] Wu H.-Y., Mao X.-F., Fan H., Li T.-F. & Wang Y.-X. p38β MAPK signaling mediates GLP-1 receptor-stimulated microglial β-endorphin expression. Submitted to Mol. Pharmacol. (2017). 10.1124/mol.116.107102 28202578

[b63] Ma W. & Quirion R. Partial sciatic nerve ligation induces increase in the phosphorylation of extracellular signal-regulated kinase (ERK) and c-Jun N-terminal kinase (JNK) in astrocytes in the lumbar spinal dorsal horn and the gracile nucleus. Pain 99, 175–184 (2002). 12237195 10.1016/s0304-3959(02)00097-0

[b64] Chauvet N. . Rat microglial cells secrete predominantly the precursor of interleukin-1beta in response to lipopolysaccharide. Eur J Neurosci 14, 609–617 (2001). 11556886 10.1046/j.0953-816x.2001.01686.x

[b65] Taves S., Berta T., Chen G. & Ji R. R. Microglia and spinal cord synaptic plasticity in persistent pain. Neural Plast 2013, 753656 (2013). 24024042 10.1155/2013/753656 PMC3759269

[b66] Kumar S., Boehm J. & Lee J. C. p38 MAP kinases: key signalling molecules as therapeutic targets for inflammatory diseases. Nature reviews Drug discovery 2, 717–726 (2003). 12951578 10.1038/nrd1177

[b67] Bachstetter A. D. . Microglial p38 alpha MAPK is a key regulator of proinflammatory cytokine up-regulation induced by toll-like receptor (TLR) ligands or beta-amyloid (A beta). J Neuroinflammation 8 (2011). 10.1186/1742-2094-8-79 PMC3142505 21733175

[b68] Tang X., Metzger D., Leeman S. & Amar S. LPS-induced TNF-alpha factor (LITAF)-deficient mice express reduced LPS-induced cytokine: Evidence for LITAF-dependent LPS signaling pathways. Proc Natl Acad Sci USA 103, 13777–13782 (2006). 16954198 10.1073/pnas.0605988103 PMC1560089

[b69] Xing B., Bachstetter A. D. & Van Eldik L. J. Inhibition of neuronal p38alpha, but not p38beta MAPK, provides neuroprotection against three different neurotoxic insults. J Mol Neurosci 55, 509–518 (2015). 25012593 10.1007/s12031-014-0372-x PMC4303701

[b70] Xing B., Bachstetter A. D. & Van Eldik L. J. Deficiency in p38beta MAPK fails to inhibit cytokine production or protect neurons against inflammatory insult in *in vitro* and *in vivo* mouse models. PloS One 8, e56852 (2013). 23457629 10.1371/journal.pone.0056852 PMC3574114

[b71] Tsuda M., Mizokoshi A., Shigemoto-Mogami Y., Koizumi S. & Inoue K. Activation of p38 mitogen-activated protein kinase in spinal hyperactive microglia contributes to pain hypersensitivity following peripheral nerve injury. Glia 45, 89–95 (2004). 14648549 10.1002/glia.10308

[b72] Schafers M., Svensson C. I., Sommer C. & Sorkin L. S. Tumor necrosis factor-alpha induces mechanical allodynia after spinal nerve ligation by activation of p38 MAPK in primary sensory neurons. J Neurosci 23, 2517–2521 (2003). 12684435 10.1523/JNEUROSCI.23-07-02517.2003 PMC6742090

[b73] Chen X. L. . Down-regulation of spinal D-amino acid oxidase expression blocks formalin-induced tonic pain. Biochem Biophys Res Commun 421, 501–507 (2012). 22521889 10.1016/j.bbrc.2012.04.030

[b74] Leitl M. D. . Pain-related depression of the mesolimbic dopamine system in rats: expression, blockade by analgesics, and role of endogenous kappa-opioids. Neuropsychopharmacology 39, 614–624 (2014). 24008352 10.1038/npp.2013.236 PMC3895239

[b75] Fan H., Li T. F., Gong N. & Wang Y. X. Shanzhiside methylester, the principle effective iridoid glycoside from the analgesic herb Lamiophlomis rotata, reduces neuropathic pain by stimulating spinal microglial beta-endorphin expression. Neuropharmacology 101, 98–109 (2016). 26363192 10.1016/j.neuropharm.2015.09.010

[b76] Gong N. . Site-specific PEGylation of exenatide analogues markedly improved their glucoregulatory activity. Br J Pharmacol 163, 399–412 (2011). 21244372 10.1111/j.1476-5381.2011.01227.x PMC3087140

[b77] Wei H. . Potential role of spinal TRPA1 channels in antinociceptive tolerance to spinally administered morphine. Pharmacol Rep 68, 472–475 (2016). 26922555 10.1016/j.pharep.2015.11.008

[b78] Zhang J. Y., Gong N., Huang J. L., Guo L. C. & Wang Y. X. Gelsemine, a principal alkaloid from Gelsemium sempervirens Ait., exhibits potent and specific antinociception in chronic pain by acting at spinal alpha3 glycine receptors. Pain 154, 2452–2462 (2013). 23886522 10.1016/j.pain.2013.07.027

[b79] Kim S. H. & Chung J. M. An experimental model for peripheral neuropathy produced by segmental spinal nerve ligation in the rat. Pain 50, 355–363 (1992). 1333581 10.1016/0304-3959(92)90041-9

[b80] Wang Y. X. & Pang C. C. Functional integrity of the central and sympathetic nervous systems is a prerequisite for pressor and tachycardic effects of diphenyleneiodonium, a novel inhibitor of nitric oxide synthase. J Pharmacol Exp Ther 265, 263–272 (1993). 7682612

